# GWAS of resistance to three bacterial diseases in the Andean common bean diversity panel

**DOI:** 10.3389/fpls.2024.1469381

**Published:** 2024-09-05

**Authors:** Alvaro Soler-Garzón, Mwiinga Mulube, Kelvin Kamfwa, Davies M. Lungu, Swivia Hamabwe, Jayanta Roy, Venâncio Salegua, Deidré Fourie, Timothy G. Porch, Phillip E. McClean, Phillip N. Miklas

**Affiliations:** ^1^ Irrigated Agriculture Research and Extension Center, Washington State University, Prosser, WA, United States; ^2^ Department of Plant Science, University of Zambia, Lusaka, Zambia; ^3^ Department of Plant Sciences, North Dakota State University, Fargo, ND, United States; ^4^ Mozambique Agricultural Research Institute (IIAM), Nampula, Mozambique; ^5^ Dry Bean Producers Organization, Pretoria, South Africa; ^6^ Tropical Agriculture Research Station, United States Department of Agriculture - Agricultural Research Service (USDA-ARS), Mayagüez, Puerto Rico; ^7^ Grain Legume Genetics and Physiology Research Unit, United States Department of Agriculture - Agricultural Research Service (USDA-ARS), Prosser, WA, United States

**Keywords:** *Phaseolus vulgaris*, halo bacterial blight, common bacterial blight, bacterial brown spot, candidate resistance genes, genome-wide association study

## Abstract

Bacterial brown spot (BBS) caused by *Pseudomonas syringae* pv. *syringae* (*Pss*), common bacterial blight (CBB) caused by *Xanthomonas axonopodis* pv. *phaseoli* (*Xap*) and *Xanthomonas fuscans* subsp. *fuscans* (*Xff*), and halo bacterial blight (HBB), caused by *Pseudomonas syringae* pv. *phaseolicola* (*Psph*), are major bacterial diseases that severely affect common bean yields and global food security. Andean-origin dry beans, representing large-seeded market classes, are particularly susceptible. Using 140,325 SNPs, a multi-locus GWAS was conducted on subsets of the Andean diversity panel (ADP) phenotyped for BBS in South Africa, CBB in Puerto Rico, South Africa, and Zambia, and HBB in South Africa, through natural infection, artificial inoculation, or both. Twenty-four QTL associated with resistance were identified: nine for BBS, eight for CBB, and seven for HBB. Four QTL intervals on Pv01, Pv03, Pv05, and Pv08 overlapped with BBS and HBB resistance. A genomic interval on Pv01, near the *fin* gene, which determines growth habit, was linked to resistance to all three pathogens. Different QTLs were detected for BBS and CBB resistance when phenotyped under natural infection versus artificial inoculation. These results underscore the importance of combining phenotyping methods in multi-GWAS to capture the full genetic spectrum. Previously recognized CBB resistance QTL SAP6 and SU91 and HBB resistance QTL HB4.2, and HB5.1, were observed. Other common (MAF >0.25) and rare (MAF <0.05) resistance QTL were also detected. Overall, these findings enhance the understanding and utilization of bacterial resistance present in ADP for the development of common beans with improved resistance.

## Introduction

The three most globally prevalent bacterial diseases in the common bean (*Phaseolus vulgaris* L.) are bacterial brown spot (BBS) caused by *Pseudomonas syringae* pv. *syringae* van Hall (*Pss*); common bacterial blight (CBB), caused by *Xanthomonas axonopodis* pv. *phaseoli* (*Xap*) and *Xanthomonas fuscans* subsp. *fuscans* Smith (*Xff*); and halo bacterial blight (HBB) caused by *Pseudomonas syringae* pv. *phaseolicola* Burkholder (*Psph*). The incidence and severity of these diseases are influenced by the plant genotype, bacterial strain, climatic factors, seed hygiene, and agricultural management practices. Under favorable conditions, these pathogens can cause up to 50% yield loss and reduce seed quality (Singh and Schwartz, 2010; Singh and Miklas, 2015; Muedi et al., 2015; Tock et al., 2017).

BBS, CBB, and HBB symptoms manifested in leaves, pods, and seeds, leading to leaf blight, sunken lesions on pods, and poor seed quality. These diseases are seed-transmitted, emphasizing the importance of disease-free seeds to mitigate their spread. While copper-based chemicals applied to growing plants can limit disease severity, planting resistant varieties is considered the most effective, economical, sustainable, and environmentally friendly approach for combatting bacterial blights in common beans (Gilbertson et al., 1992; Yu et al., 2012). However, breeding for resistance to bacterial blight diseases in common bean is difficult because the resistance is inherited in a complex fashion, and the methods available for screening germplasm are cumbersome.

BBS resistance in common beans is quantitatively inherited by multiple factors that influence infection. Antonius (1982) observed that multiple recessive genes conditioned the resistance to BBS. Resistance to BBS exhibits low heritability when measured under field conditions relative to greenhouse inoculations, and quantitative trait loci (QTL) studies conducted by Jung et al. (2003) in dry bean and Navarro et al. (2007) in snap bean, using recombinant inbred populations, corroborate the complexity and quantitative inheritance of resistance to BBS across multiple screening methods and environments. Tropical black beans A55 and ‘Puebla-152’ of Middle American origin were the sources of resistance for the dry and snap bean studies, respectively. Although breeding for BBS in snap beans has been a focus (Navarro et al., 2007), efforts to incorporate such resistance into large-seeded Andean dry beans are unknown.

Screening bean lines for reactions to BBS is difficult, in part, because natural infection is dependent on the population size of *Pss* on leaves. Natural infections are also triggered by environmental events such as rainfall, hail, and wind. *Pss* survives on healthy bean leaves as an epiphyte until favorable environmental conditions lead to a substantially increased population size, inducing infection (Hirano et al., 1987). This suggests that genetic factors that lower the *Pss* population size may reduce the incidence of BBS disease. CBB resistance has been observed in various common bean gene pools. From the tertiary gene pool, high levels of resistance have been detected in tepary bean (*Phaseolus acutifolius*) (Singh and Muñoz, 1999). Moderate resistance in the common bean has been obtained from interspecific hybridization with *Phaseolus coccineus* from the secondary gene pool (Welsh and Grafton, 2001). *P. vulgaris* sources of CBB resistance include the Montana #5 (Miklas et al., 2003) and Thlantaplanta 46 (PI 208762) landraces. The genetic control of CBB disease resistance in the common bean is complex, with more than 20 QTL for resistance detected across all 11 chromosomes (Singh and Miklas, 2015; Singh et al., 2001). The major QTL on Pv06 (BC420), Pv08 (SU91), and Pv10 (SAP6) have been well characterized, but none are completely resistant to aggressive *Xap* or *Xff* strains (Miklas et al., 1996; Pedraza et al., 1997; Yu et al., 1998; Vandemark et al., 2008). Traditional marker-assisted selection (MAS) has been used to develop large-seeded Andean beans that are resistant to CBB (Miklas et al., 2006a, 2006b; Viteri et al., 2014).

Resistance to HBB is conditioned by major R genes (*Pse-1*, *Pse-2*, *Pse-3*, *Pse-4*, and *Pse-6*), with differential reactions across a set of nine *Psph* race differentials (Taylor et al., 1996a; Yaish et al., 2006; Miklas et al., 2014; Trabanco et al., 2014). QTL HB4.2 is critical for the control of the globally prevalent Race 6. In CAL 143, a large-seeded red mottled CIAT breeding line, released as a cultivar in several East African countries, the HB4.2 QTL conditions resistance to Race 6 derived from PI 150414, a small red landrace from El Salvador (Tock et al., 2017). GWAS analysis of ADP by Tock et al. (2017) revealed only one QTL, HB5.1, which correlated with higher yield under Race 6 infection. An Andean red bean, called ‘Rojo,’ released by Sokoine University of Agriculture (SUA), possesses *Pse-2*, a major gene that provides resistance to seven of nine differential races but not Race 6.

To facilitate breeding for resistance to bacterial diseases in Andean beans, we used multilocus GWAS methods to identify and compare genomic regions and candidate genes significantly associated with resistance to BBS, CBB, and HBB diseases in a large diversity panel of Andean accessions tested under controlled and natural inoculations.

## Materials and methods

### Plant material

A *P. vulgaris* Andean diversity panel (ADP, n = 468) was established to enhance the genetic improvement of beans from the Andean gene pool (Cichy et al., 2015). ADP includes a diverse range of landraces, germplasm releases, advanced breeding lines, and released cultivars from Africa, the Americas (Central, North, and South), Asia, the Caribbean, and Europe (**Supplementary Table S1**). In this study, ADP was expanded using 109 additional accessions.

### Phenotyping

#### Bacterial brown spot

A total of 378 ADP accessions and five check cultivars were previously evaluated for phenotypic disease reactions to BBS using an alpha-lattice design with three replications at three field locations in South Africa (Middleburg, Potchefstroom, and Warden) (Salegua et al., 2020) (**Supplementary Table S2**). The Middelburg and Warden trials were conducted under natural infection, and artificial inoculation was used to enhance infection in Potchefstroom. The inoculum consisted of three aggressive *Pss* isolates (BV 6.3, BV 3.3.2, and BV 27.1) suspended in 1 × 10^8^ CFU/mL and applied using a mist blower with supplemental irrigation to enhance infection (Muedi et al., 2015). BBS severity was evaluated 7 days after the first signs of infection and then weekly for 3 weeks using the 1 to 9 CIAT scale, where 1 = no symptoms, 5 = moderate disease symptoms, and 9 = severe disease symptoms and plant death (Van Schoonhoven and Pastor-Corrales, 1987). A similar 1 to 9 scale was used to evaluate disease severity in the CBB and HBB trials, respectively. For all three diseases, the disease scores were grouped into three categories: 1–3 for resistant plants with no discernible or few symptoms with little impact on yield, 4–6 for plants with moderate disease symptoms with some impact on seed yield, and 7–9 for susceptible plants with severe symptoms or plant death. The mean disease severity scores for BBS recorded 21-d after inoculation were used for GWAS. Note that the BBS reaction data collected from the Warden field location were excluded from this study because of the confounding influence of additional stress factors.

#### Common bacterial blight

The reaction of ADP with CBB has not been reported previously. Consequently, various subsets of ADP accessions were screened against specific *Xap* strains in the greenhouse and against endemic strains in the field. The ADP accessions (n = 249) were inoculated with *X. axonopodis* pv. *phaseoli* (*Xap*) strains Xa3353 and Xa484A in a screenhouse at the USDA-ARS Tropical Agriculture Research Station in Mayagüez, Puerto Rico, in 2012 (**Supplementary Table S2**). The inoculum was produced on yeast extract-dextrose-CaCO_3_ agar (YDCA) for 48 h and then diluted in sterile water to 10^7^ CFU/mL (Zapata et al., 1985). The multiple-needle inoculation technique, first described by Andrus (1948), consists of approximately 18 pins arranged in a 2.5 cm diameter that are pressed against the fully expanded first trifoliate leaves (~two weeks after planting) that are backed by sponges soaked in inoculum. Three replicates in a randomized complete block design (RCBD) were tested with a susceptible control, ‘Morales,’ and a resistant control, VAX 6. Plants were rated (1–9) for disease severity 21 days after inoculation.

A set of 193 ADP accessions were screened for resistance to *Xanthomonas fuscans* subsp. *fuscans* (*Xff*) strain ZM4 by artificial inoculation in the screenhouse at the Agricultural Research Council-Grain Crops Institute (ARC-GCI) located in Potchefstroom, South Africa (**Supplementary Table S2**). The ZM4 strain was isolated from a CBB-infected leaf sample collected in 2018 in a common bean field in Lusaka, Zambia. Fourteen to twenty-day-old plants were inoculated using the multiple-needle inoculation method described above. Plants were maintained in a screenhouse at 28°C day/18°C night temperatures. Plants were rated for disease severity 14 days post-inoculation. The experimental design was RCBD with three replicates.

A set of 201 ADP accessions were tested under natural field infection at the Zambia Agricultural Demonstration Center at the University of Zambia in Lusaka, Zambia (**Supplementary Table S2**). The panel entries ADP665 (USWK-CBB-17) and ADP676 (CELRK) were used as resistant and susceptible checks, respectively. The trial was conducted in December 2013, with two replications in an RCBD. The plot size was a single row 4 m long, with an inter-row spacing of 0.5 m. Common bacterial blight (CBB) disease is caused by the *X. axonopodis* pv. *phaseoli* and was prevalent at the site where ADP was planted. The University of Zambia experimental site is located in a region that tends to have high CBB pressure because of higher temperatures and rainfall during the growing season from January to April. The early 2014 season was no exception. Disease pressure was uniform across the experimental field, as reflected by the consistent scores obtained for the resistant and susceptible checks in both replications. CBB reactions at the flowering and pod-filling growth stages were evaluated under natural infection conditions using a previously described disease severity scale.

In addition, 255 ADP accessions were evaluated for CBB resistance under natural infection in the field at the University of Zambia Research Farm in Lusaka, Zambia in 2018 (**Supplementary Table S2**). Evaluations were conducted during the rainy season on soil classified as fine loamy isohyperthermic paleustalf. The experimental design and plot size were the same as those used for 2014. A local landrace from Zambia, ‘Kabulangeti’, which is highly susceptible to CBB, was used as a susceptibility check. The disease pressure was uniform across the experimental field, as reflected by the consistently high CBB severity scores for ‘Kabulangeti’ within and across replications. The CBB severity was scored for each plot at the pod-filling stage. For every CBB trial, least-square mean disease severity scores were used for GWAS.

#### Halo bacterial blight

ADP accessions (n = 360) were phenotyped for reactions (1 to 9 scale) to halo blight under field conditions at the Potchefstroom ARC-GCI Station in South Africa (Tock et al., 2017) (**Supplementary Table S2**). Briefly, the trial was sown in single-row plots of 4 m length per line, spaced 0.75 m apart, and replicated three times in an RCBD. The halo blight host differential set (Taylor et al., 1996b) was included as a check. The *Phps* inoculum consisting of Race 6 (Tanzania-1299A strain) was cultivated in King’s B medium, suspended in water, adjusted to 108–109 CFU ml^−1^, and applied using a mist-blower, followed by supplemental irrigation to enhance disease development. Disease severity (1–9 scale) was assessed during the mid-pod-fill stage. The mean disease severity scores obtained by Tock et al. (2017) were used for the GWAS in this study.

Pearson correlations between mean bacterial disease scores were performed using the *cor()* function in R 4.4.0 (R Core Team, 2021) with the parameter *use = “pairwise.complete.obs.”* Visualization and clustering of the correlation coefficients were performed using the R package d3heatmap (https://github.com/erdogant/d3heatmap).

### Genotyping

Fresh young leaves from the early trifoliate stage of a single plant were collected and genomic DNA was extracted using the Mag-Bind Plant DNA Plus Kit (Omega Bio-Tek, Norcross, Georgia, USA). Genomic libraries were constructed using the optimized two-enzyme protocol [MseI and Taqα1] developed by Schröder et al. (2016). The libraries were sequenced using two different methods at the Hudson Alpha Institute for Biotechnology, Huntsville, AL. DNA from the first set, consisting of 325 ADP genotypes, was sequenced in single-end runs using the Illumina HiSeq 2500 sequencing platform. A total of 228 genotypes were included in the second set and library sequencing was performed using the Illumina HiSeq 2500 sequencing system and paired-end runs (2 × 150 bp). In the second set, 53 sequenced genotypes were duplicated from the first set of sequenced genotypes. Duplicated, Middle-American, and Tepary genotypes were removed, resulting in a total of 468 ADP genotypes.

Raw fastq files were subjected to quality filtering using the SICKLE software (Joshi and Fass, 2011) to trim reads with a low-quality score ≤20 and a minimum of 80 bp in length. The processed reads (forward reads from the paired-end reads) were aligned against the common bean reference genome G19833*v2.1* (https://phytozome-next.jgi.doe.gov/info/Pvulgaris_v2_1) (Schmutz et al., 2014) using the Burrows–Wheeler Alignment Tool (BWA-mem) with default parameters (Li, 2013). SAMtools *v1.15.1* (Li et al., 2009) was used to sort and index the aligned mapping results. Read group information of corresponding genotypes was added using Picard *v2.9.0* tools (http://broadinstitute.github.io/picard).

Finally, the *MultisampleVariantsDetector* module embedded in NGSEPcore_4.2.0 software was used to call the variants with the ‘*-maxAlnsPerStartPos* 100’ parameter (Tello et al., 2023). Multiallelic SNPs were discarded, and markers with minDP ≥3, a quality value of >40, less than 40% missing sites, and 1% minor allele frequency (MAF) were selected, resulting in 184,345 high-quality SNPs genotyped in the 468 ADP accessions. The selected SNPs were imputed using the *VCFImpute* module in NGSEP v4.2.0 (Tello et al., 2023). This module utilizes a Hidden Markov Model (HMM) implemented in the fastPHASE package (Scheet et al., 2006).

### ADP population structure

Population structure analysis was performed using 468 ADP accessions genotyped with an unimputed subset of 2,568 SNPs after linkage disequilibrium (LD) pruning, which involved using a 0.5 *R^2^* threshold and the *–indep-pairwise* function in Plink v2.0 (https://www.cog-genomics.org/plink/2.0/) (Purcell et al., 2007), with a sliding window of 50 kb, shifted by five bases. The pruned set of SNPs with less than 20% missing values and a MAF >0.01 were retained for genetic structure analysis.

Population structure was estimated using a Bayesian Markov chain Monte Carlo model (MCMC) implemented in STRUCTURE 2.3 software (Pritchard et al., 2000). Ten runs were performed for each population (k), which were set from 1 to 10. The burn-in time and MCMC replication number were set as 100,000 and 200,000, respectively. The most likely number of populations (k) in the ADP was calculated using the Evanno method (Evanno et al., 2005) implemented in Pophelper v2.3.1 (Francis, 2017). To determine the robustness of the assignments of individuals to populations at each K, the Q-matrix was obtained using CLUMPP v1.1 software (Jakobsson and Rosenberg, 2007). Finally, the filtered SNPs were utilized for PCA and kinship analyses.

### Genome-wide association study

Twenty-two Middle-American and two admixture accessions identified in the structure analysis were excluded, resulting in 444 genotypes analyzed with 148,701 SNPs filtered using previously described parameters. An LD-pruned set of 2,136 SNPs was obtained for principal components analysis (PCA) (**Supplementary Table S3**) and an identity-by-state kinship matrix, calculated using the Zhang algorithm. Both analyses were performed using GAPIT *v3.0* R package (Wang and Zhang, 2021). Additionally, a multi-locus GWAS (ML-GWAS) analysis was performed using the mrMLM *v4.0* R package (Zhang et al., 2020), which includes six different statistical methods for traits with multiple and polygenic effects: mrMLM, FASTmrMLM, FASTmrEMMA, EM-BLASSO, pLARmEB, and pKWmEB (Wang et al., 2016; Wen et al., 2018). To correct any genetic errors and prevent false discoveries, the kinship matrix (K) and first three principal components (PCs) were used as covariates. A logarithm of the odds (LOD) score of 3.0 (or *P* = 0.0002) was employed as a cutoff in ML-GWAS to strike a balance between high power and low false-positive rate for detecting QTL, as recommended by Zhang et al. (2019). Significant QTL detected by ML-GWAS were plotted using the Circlize R package (Gu et al., 2014).

Furthermore, analysis of variance comparing groups based on the presence or absence of favorable quantitative trait loci (QTL) per bacterial trait against phenotypic means was conducted using Yuen’s test for trimmed means. This analysis was performed using the *ggbetweenstats* function in the ggstatsplot R package. Yuen’s test was applied using the robust *p-value* method (*P* ≤0.05) within the same ggbetweenstats function (Patil, 2021). Only QTL identified as statistically stable (LOD >3) by at least two or more ML-GWAS methods combined with significant (*P* ≤0.05) Yuen’s tests were considered to harbor favorable alleles.

### Candidate genes

The genome browser (JBrowse) in Phytozome 13 was used to search the G19833*v2.1* common bean genome and identify positional candidate genes associated with significant SNPs. A gene was considered a candidate if it contained a significant SNP or was in the same LD block as the most significant SNP. Furthermore, a potential candidate gene is required to encode a protein with an established or proposed role in disease resistance or as homologs of guardees and decoys involved in bacterial resistance in other species (Kourelis and van der Hoorn, 2018).

Haplotype-based GWAS data were analyzed using the Plink software package. Haplotype blocks were constructed using the following parameters: *–blocks no-pheno-req*, *–blocks-max-kb 1000*, *–blocks-min-maf 0.05*, *–blocks-strong-lowci 0.70*, *–blocks-strong-highci 0.98*, *–blocks-recomb-highci 0.90*, and *–blocks-inform-frac 0.95*.

## Results

### ADP diversity analysis

**Figure 1:**
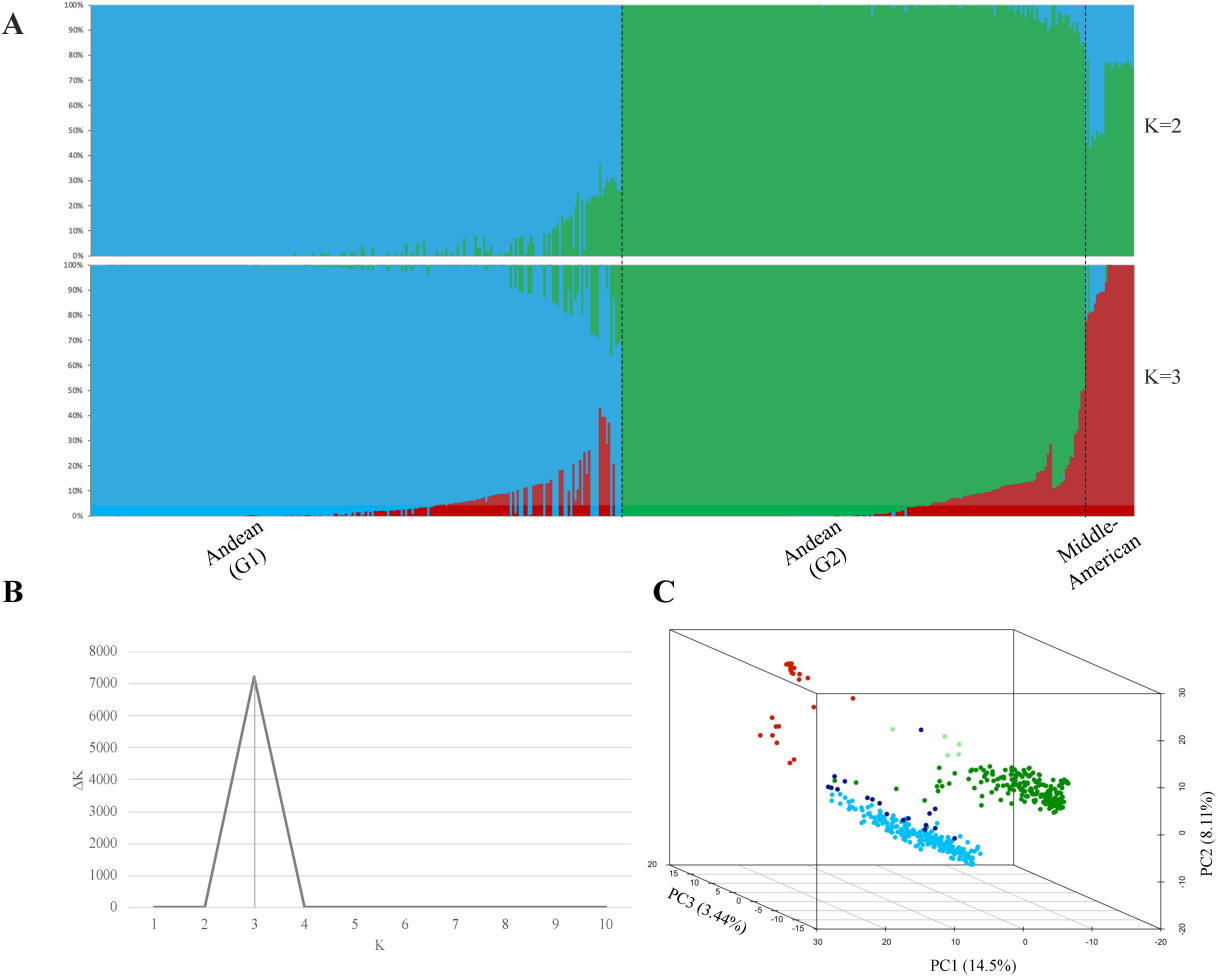
Estimation of the population structure. **(A)** Population structure clustering depicts each of the 468 ADP accessions by vertical bars into colored segments, with lengths proportional to each of the K inferred ancestral populations: blue and green colors represent sub-populations G1 and G2, respectively, within the Andean gene pool, while the red color corresponds to the Middle-American Genepool. **(B)** Evanno test of ADP populations resulting in an optimal K = 3, and **(C)** Principal Component Analysis (PCA) of the Andean diversity panel of common bean, with colors based on population structure analysis at K = 3, based on the membership coefficient (Q ≥0.75) from STRUCTURE. In the plot, Middle-American population is represented in red color, Andean (G1) population in blue, Andean (G2) admixture accessions in ‘light green,’ Andean (G2) population in green, and Andean (G1) admixture accessions in ‘dark blue’.

Structural analysis of 468 ADP accessions genotyped with 2,658 linkage disequilibrium-pruned SNP markers resulted in the separation of three subpopulations (K = 3) based on an optimal K determined by the Evanno test (**Figures 1A, B**). Accessions were categorized based on a membership coefficient (Q) greater than 0.75 at K = 2, the ADP accessions were divided into two subpopulations: Andean (G1) and another subpopulation that combined Andean (G2) and Middle-American accessions. At K = 3, the Andean (G2) and Middle-American subpopulations were separated according to Q ≥0.75. Additionally, the PC analysis carried out for GWAS confirmed the subpopulations described above for the same set of ADP accessions. The percentage of genetic diversity explained by each of the three PCs was 14.5% for PC1, 8.11% for PC2, and 3.44% for PC3 (**Figure 1C**).

Additionally, 22 accessions categorized into the Middle-American gene pool (Q ≥0.75) and two accessions with a Middle-American allele admixture (Q >0.5) were removed from further analyses. This group included five Mexican accessions of the M phaseolin type, which is predominant in Mexican wild bean accessions (Koenig et al., 1990).

In summary, the Andean population comprised 444 accessions with structural analysis separating them into two sub-populations: Andean (G1) with 221 accessions and Andean (G2) with 202 accessions. Andean (G1) accessions were predominantly from Africa (55.7%, n = 123), followed by those of North American origin (32.1%, n = 71). Similarly, the Andean (G2) accessions included a significant proportion from Africa (42.6%, n = 86) and North America (20.8%, n = 42), with a notable representation from South America (23.3%, n = 47). The remaining accessions were grouped as admixtures according to gene-pool predominancy, using a membership coefficient Q ≥0.5, with 17 accessions as Andean (G1) admixture (3.82%) and four as Andean (G2) admixture (0.90%).

### Phenotypic correlations

**Figure 2:**
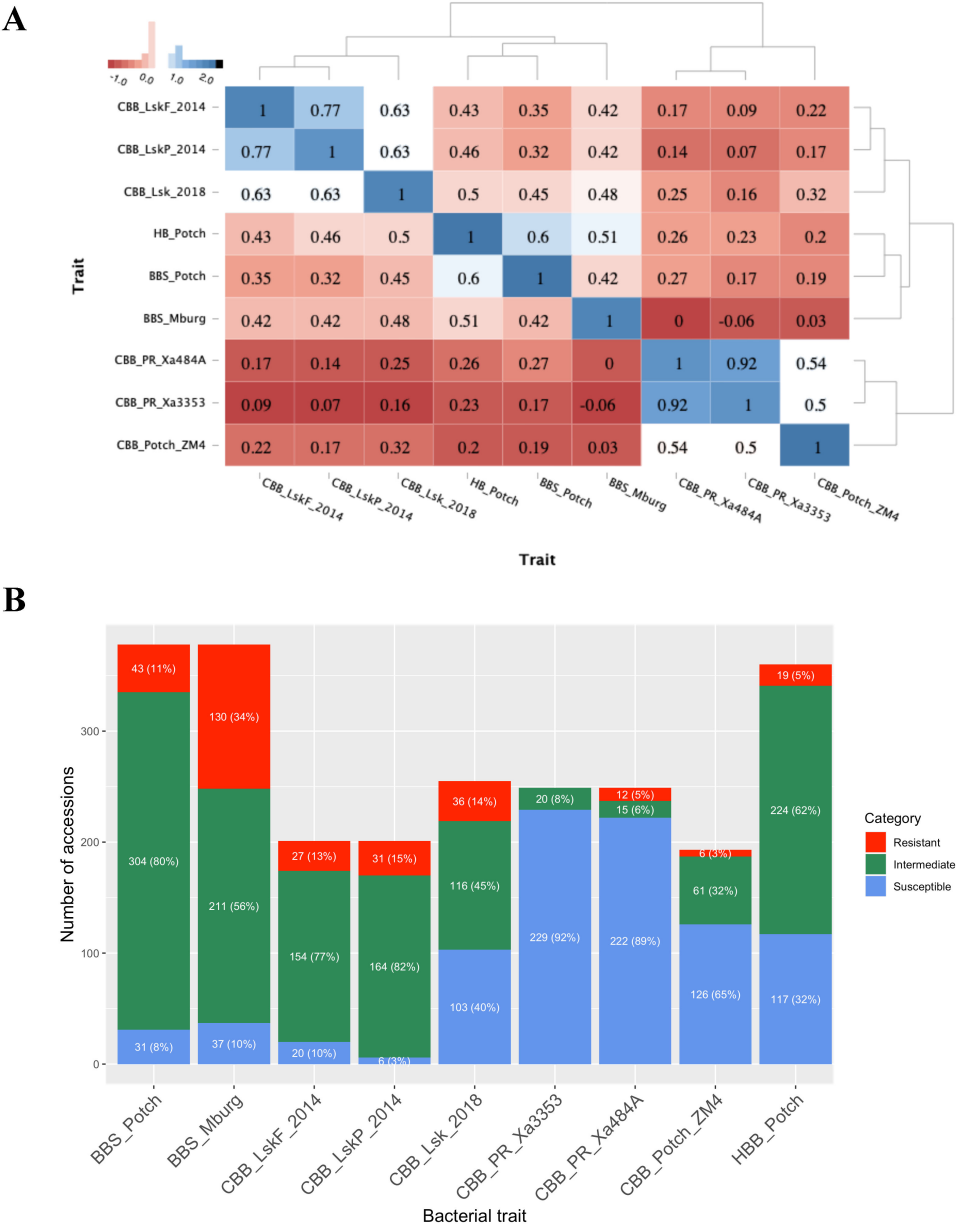
Bacterial phenotyping analysis. **(A)** Dendrogram and heatmap based on the Pearson correlation coefficient between traits and locations, using the pairwise complete observation method; **(B)** Frequency distribution of the 1–3 resistant (red), 4–6 intermediate (green), and 7–9 susceptible (blue) disease score categories for each trial.

Based on heatmap analysis, three clusters were observed using phenotypic data collected for BBS, CBB, and HBB reactions in ADP (**Figure 2A**). One cluster grouped CBB field reactions collected in Lusaka, Zambia in 2014 and 2018. The second cluster grouped BBS and HBB reaction data. The third cluster consisted of CBB screenhouse reactions collected in Puerto Rico using the Xa484A and Xa353 strains in 2012 and the ZM4 strain evaluated in Potchefstroom, South Africa, in 2018.

The CBB data collected at the flowering and pod-filling stages in Lusaka, Zambia, in 2014 were highly correlated (*r* = 0.77, *P <*0.0001), and correlated with the data collected in Lusaka in 2018 (*r* = 0.63, *P <*0.0001). The screenhouse CBB data collected in Puerto Rico using Xa353 and Xa484A strains was highly correlated (*r* = 0.92, *P <*0.0001) and moderately correlated, 0.50 (*P <*0.0001) and 0.54 (*P <*0.0001), respectively, with the CBB screenhouse data collected using the ZM4 strain in Potchefstroom, South Africa, in 2018.

BBS reactions collected in Potchefstroom and Middelburg exhibited a correlation of 0.42 (*P <*0.0001). BBS reactions obtained in Potchefstroom and Middelburg were moderately correlated with HBB reaction, 0.60 (*P <*0.0001) and 0.51 (*P <*0.0001), respectively. BBS reactions from Potchefstroom and Middelburg exhibited moderate correlations ranging from 0.32 (*P <*0.0001) to 0.48 (*P <*0.0001), with field reactions to CBB obtained in Lusaka in 2014 and 2018. Only BBS reactions from Potchefstroom exhibited low but significant correlations, with CBB reactions collected in the screenhouse ranging from 0.17 (*P <*0.05) to 0.27 (*P <*0.0001). Lastly, HBB reactions showed significantly moderate correlations with CBB reactions collected in Lusaka (0.43 to 0.59; P <0.0001) and low but significant correlations [0.20 (*P <*0.01) to 0.26 (*P <*0.001)] with CBB reactions from screenhouse trials in Puerto Rico and South Africa.

Based on the resistant (1–3), intermediate (4–6), and susceptible (7–9) disease score categories, more resistant individuals were detected under natural field infection than under artificial inoculation in the field or screenhouse (**Figure 2B**). Accessions evaluated using artificial inoculation with CBB Xa3353, Xa484A, and ZM4 strains demonstrated high susceptibility rates of 92%, 89%, and 65%, respectively. Conversely, intermediate scores were predominant for the accessions evaluated for CBB under natural infection conditions in Lusaka in 2014 and 2018. Similarly, the BBS and HBB evaluations showed predominantly intermediate scores ranging from 56% to 82%.

### Genome-wide association study

**Figure 3:**
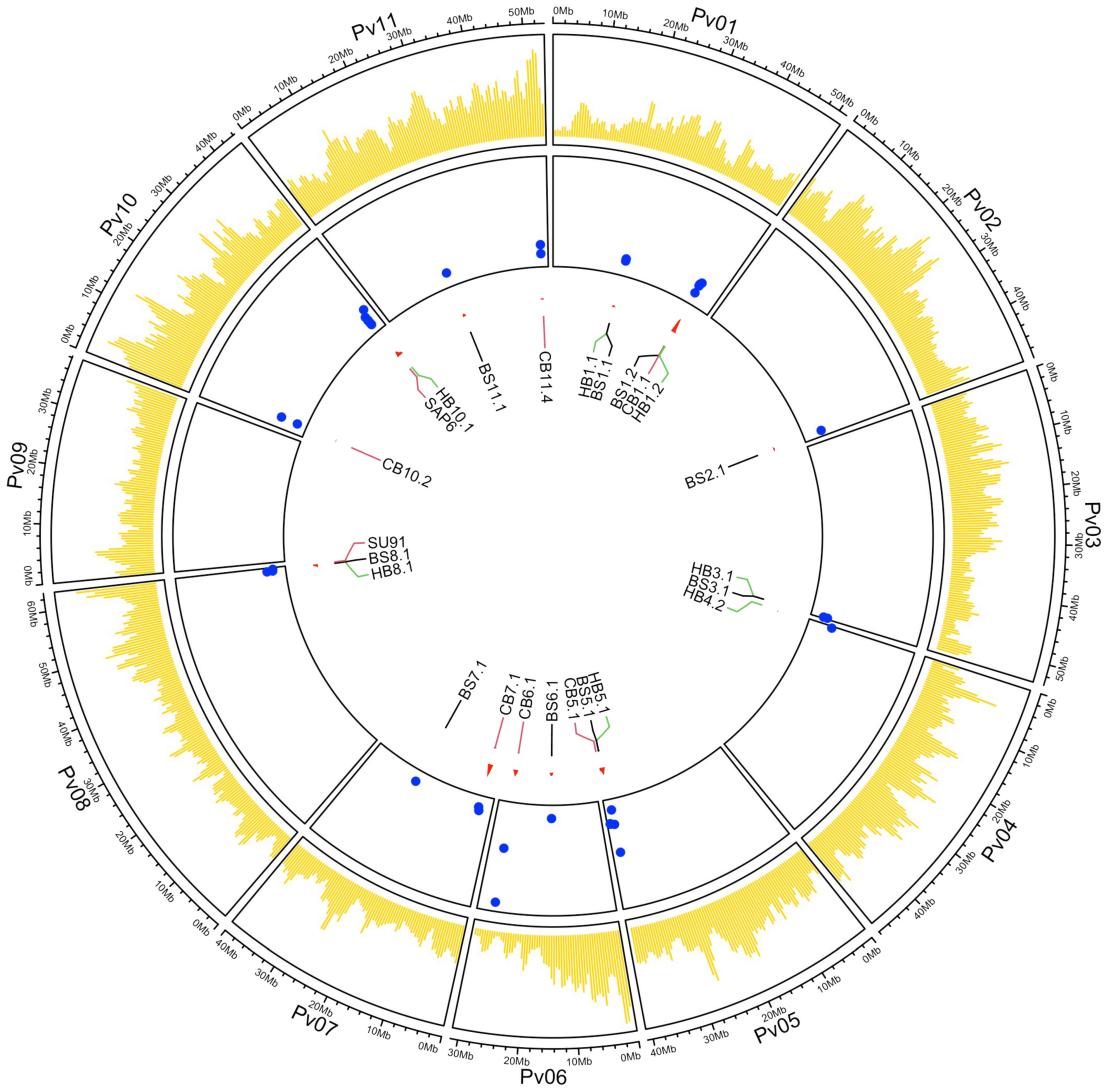
Circos plot showing the SNP distribution map for the 444 ADP genotypes in yellow, LOD values (blue dots on the y-axis), and genomic positions for the labeled significant QTL (on the x-axis) associated with BBS, CBB, and HBB resistance across 11 chromosomes. The SNPs were positioned using the G19833 *v2.1* reference genome assembly.

A GWAS detected 24 significant QTL associated with resistance to these three bacterial diseases. Significance (LOD >3) in two or more ML-GWAS methods combined with significant (*P* ≤0.05) Yuen’s tests were used to declare a significant QTL in this study. The QTL were distributed across 10 of the 11 chromosomes, with Pv09 being the exception (**Figure 3**). The QTL for each disease are described below.

### Bacterial brown spot QTL

Nine QTL associated with BBS resistance were identified. Four QTL (BS1.2, BS7.1, BS8.1, and BS11.1) were identified only in the Middelburg field trial under natural infection, four QTL (BS1.1, BS2.1, BS3.1, and BS6.1) were identified only in the Potchefstroom field trial under artificial inoculation, and only one, BS5.1, identified in both trials (**Table 1**). BS1.2 was detected by most of the GWAS methods (six), followed by BS1.1 and BS11.1 (five methods). BS2.1 exhibited the lowest MAF (0.09), whereas BS5.1 exhibited the highest MAF (0.43, 0.45). Interestingly, BS5.1 had a greater effect in Potchefstroom, with 6.25% phenotypic variation explained (PVE), than in Middelburg, with 2.80% PVE.

**Table 1:** BBS QTL identified by ML-GWAS in 377 ADP accessions evaluated against *Pseudomonas syringae* pv. *syringae* under natural field infection in Middelburg (Mburg) and artificial field inoculation in Potchefstroom (Potch).

Trait name	QTL	Method^a^	Chr.	Peak position (bp)	Block LD min	Block LD Max	QTL effect	LOD score	PEV (%)^b^	MAF^c^
BBS_Potch	BS1.1	2, 3, 4, 5, 6	Pv01	21,832,490	21,793,125	21,846,851	0.60	6.36	6.31	0.12
BBS_Mburg	BS1.2	1, 2, 3, 4, 5, 6	Pv01	44,808,951	44,576,796	44,854,819	0.53	8.06	6.54	0.31
BBS_Potch	BS2.1	3, 5	Pv02	47,980,026	47,976,073	48,027,062	0.46	6.87	5.87	0.09
BBS_Potch	BS3.1	1, 2, 5, 6	Pv03	53,107,156	53,080,736	53,156,072	−0.34	4.86	3.32	0.37
BBS_Mburg	BS5.1	4, 5	Pv05	39,065,428	38,872,419	39,106,281	−0.26	4.11	2.80	0.43
BBS_Potch	BS5.1	2, 5, 6	Pv05	39,065,428	38,872,419	39,106,281	−0.32	9.25	6.25	0.45
BBS_Potch	BS6.1	1, 2, 5, 6	Pv06	14,661,470	14,648,598	14,741,327	0.28	4.97	5.43	0.28
BBS_Mburg	BS7.1	4, 5, 6	Pv07	24,538,230	24,537,459	24,538,340	−0.52	4.43	9.44	0.17
BBS_Mburg	BS8.1	2, 6	Pv08	62,072,813	62,020,853	62,128,602	0.31	4.91	3.20	0.28
BBS_Mburg	BS11.1	2, 3, 4, 5, 6	Pv11	22,627,255	22,593,204	22,674,604	−0.52	5.37	7.29	0.26

^a^mrMLM, FASTmrMLM, FASTmrEMMA, pLARmEB, pKWmEB, and EM-BLASSO were indicated by 1–6, respectively. ^b^PVE (%), percentage of phenotypic variation explained by each QTp. ^c^MAF, minor allele frequency determined from the peak significant SNP.

Three ADP accessions, ADP118 (‘Werna’), ADP120 (‘Tygerberg’), and ADP121 (‘Kranskop HR-1’), all indeterminate vine sugar/cranberry cultivars developed by the ARC-Grain Crops Institute, Potchefstroom, SA, possessed the same haplotype with seven QTL, BS1.1/BS1.2/BS5.1/BS6.1/BS7.1/BS8.1/BS11.1, for resistance to BBS. These three cultivars exhibited resistant disease scores ranging from 1 to 1.67 under artificial inoculation, and from 2 to 2.67 under natural infection (**Supplementary Table S2**). Additionally, two advanced lines, ADP740 (MW-25) from CIAT-Malawi and ADP790 (PR0633-10) from Puerto Rico, with BS1.1/BS1.2/BS6.1/BS7.1/BS8.1/BS11.1 and BS1.1/BS1.2/BS6.1/BS8.1/BS11.1, respectively, exhibited low disease scores (ranging from 1 to 1.67) across locations.

### Common bacterial blight QTL

Eight QTL, CB1.1, CB5.1, CB6.1, CB7.1, CB10.2, and CB11.4, including the historical major QTL SAP6 and SU91, were detected across CBB trials (**Table 2**). The SAP6 QTL was detected in all trials, except for the flowering stage evaluation in Lusaka in 2014. CB1.1, CB7.1, and SU91 were only detected in Lusaka field trials. Conversely, CB5.1, CB6.1, CB10.2, and CB11.4 were only detected by the two strains in the Puerto Rico screenhouse trial, and they possessed extremely low MAF ranging from 0.01 to 0.04. The screenhouse trial in Potchefstroom detected only the SAP6 QTL.

**Table 2:** CBB QTL identified by ML-GWAS in 191–254 ADP accessions evaluated under natural infection in the field in Lusaka, Zambia (CBB_LskF_2014, CBB_LskP_2014, and CBB_Lsk_2018), and against *Xanthomonas axonopodis* pv. *phaseoli* (*Xap*) strains Xa3353 and Xa484A in the screenhouse in Mayaguez, Puerto Rico (CBB_PR_Xa3353) and by *Xanthomonas fuscans* subsp. f*uscans* strain ZM4 in the screenhouse in Potchefstroom, South Africa (CBB_Potch_ZM4).

Trait name	QTL	Method^a^	Chr.	Peak position (bp)	Block LD min	Block LD Max	QTL effect	LOD score	PEV (%)^b^	MAF^c^
CBB_LskF_2014	CB1.1	1, 2, 4, 5, 6	Pv01	44,808,951	44,576,796	44,854,819	0.47	7.37	7.94	0.23
CBB_LskP_2014	CB1.1	1, 2, 3, 4, 5, 6	Pv01	44,808,951	44,576,796	44,854,819	0.44	4.59	5.25	0.23
CBB_Lsk_2018	CB1.1	3,6	Pv01	44,808,951	44,576,796	44,854,819	0.62	7.70	5.95	0.23
CBB_PR_Xa3353	CB5.1	1, 2, 6	Pv05	40,128,807	40,044,505	40,129,080	0.94	8.92	6.40	0.02
CBB_PR_Xa484A	CB5.1	1, 2, 4	Pv05	40,128,807	40,044,505	40,129,080	1.43	8.72	9.61	0.02
CBB_PR_Xa3353	CB6.1	2, 6	Pv06	27,381,648	27,374,967	27,581,765	1.74	35.65	7.89	0.01
CBB_PR_Xa484A	CB6.1	2, 4, 6	Pv06	27,381,648	27,374,967	27,581,765	2.09	16.63	6.33	0.01
CBB_LskF_2014	CB7.1	1, 6	Pv07	3,855,073	3,764,305	3,983,142	−0.41	5.60	6.43	0.32
CBB_LskP_2014	CB7.1	1, 4, 5	Pv07	4,175,098	4,095,723	4,267,674	0.29	4.39	5.89	0.48
CBB_LskF_2014	SU91	1, 4	Pv08	62,538,816	62,534,585	62,555,715	1.12	4.79	6.46	0.03
CBB_PR_Xa3353	CB10.2	2, 4, 5, 6	Pv10	3,151,971	3,142,065	3,156,781	0.62	9.74	5.33	0.04
CBB_PR_Xa484A	CB10.2	2, 5	Pv10	3,151,971	3,142,065	3,156,781	0.63	3.72	8.33	0.04
CBB_LskP_2014	SAP6	1, 2, 6	Pv10	41,066,486	40,991,447	41,152,975	0.31	3.55	2.72	0.18
CBB_PR_Xa3353	SAP6	1, 2, 5, 6	Pv10	41,077,539	40,991,447	41,152,975	0.32	5.46	4.26	0.15
CBB_PR_Xa484A	SAP6	4, 5	Pv10	41,077,539	40,991,447	41,152,975	0.43	4.41	15.23	0.15
CBB_Lsk_2018	SAP6	4,5	Pv10	41,077,539	40,991,447	41,152,975	0.61	6.87	5.62	0.16
CBB_Potch_ZM4	SAP6	4, 6	Pv10	41,077,539	40,991,447	41,152,975	0.60	5.51	7.44	0.16
CBB_PR_Xa3353	CB11.4	1,6	Pv11	51,458,769	51,417,348	51,459,029	1.10	8.10	6.54	0.02
CBB_PR_Xa484A	CB11.4	1,6	Pv11	51,458,769	51,417,348	51,459,029	1.68	4.94	6.78	0.02

^a^mrMLM, FASTmrMLM, FASTmrEMMA, pLARmEB, pKWmEB, and EM-BLASSO were indicated by 1–6, respectively. ^b^PVE (%), percentage of phenotypic variation explained by each QTL. ^c^MAF, minor allele frequency determined from the peak significant SNP.

ADP626 (‘Badillo’), ADP113 (‘OPS-RS4’), and ADP118 (Werna) exhibited low mean CBB disease scores across the trials. ADP626, an indeterminate light red kidney from UPR-Mayaquez, Puerto Rico, possessed the CB1.1/CB3.1/CB5.1/SAP6 haplotype and exhibited a mean score of 1.3 for field locations in Zambia with natural infection, and a score of 4.2 across screenhouse trials (**Supplementary Table S2**). ADP113 and ADP118, both indeterminate sugar/cranberry cultivars from ARC-Grains Crop Institute, possessed haplotype CB1.1/SU91/SAP6 and exhibited mean scores of 1.3 and 1.0 under natural infection and 4.7 and 3.6 under artificial inoculation, respectively.

Additionally, ADP653 (USDK-CBB-15) (Miklas et al., 2006a) and ADP665 (USWK-CBB17) (Miklas et al., 2006b) released by USDA-ARS Prosser, WA, possessed haplotype SU91/SAP6 as expected and exhibited mean scores of 2.7 and 2.8, respectively, under natural infection, but were not included in the screenhouse trials. ADP653, a dark red kidney, and ADP665, a white kidney, have determinate bush growth habits.

### Halo bacterial blight QTL

Seven QTL associated with HBB resistance HB1.1, HB1.2, HB3.1, HB4.2, HB5.1, HB8.1, and HB10.1 were detected (**Table 3**). HB4.2, identified by Tock et al. (2017) in multiple populations, had a low MAF of 0.03 in this study, as expected, given its origin from the Middle American landrace PI 150414. Using ADP means, Tock et al. (2017) identified only one QTL, HB5.1. Improved GWAS methods and the enhanced SNP dataset likely contributed to the identification of six more QTL in this study. HB5.1 exhibited the highest PVE (9.73%) and LOD (19.1) and a high MAF (0.44), which is significantly consistent with the MAF observed (MAF = 0.417) by Tock et al. (2017). HB8.1, was observed by Tock et al. (2017) in an RIL population, and HB10.1 is near the *Pse-4* gene locus (Miklas et al., 2014); however, these and the newly identified QTL HB1.1, HB1.2, and HB3.1, require further validation.

**Table 3:** HBB QTL identified by ML-GWAS in 358 ADP accessions inoculated with *Pseudomonas syringae* pv. *phaseolicola* Race 6 (strain Tanzania-1299A) under field conditions at the Potchefstroom ARC-GCI Station in South Africa.

Trait name	QTL	Method^a^	Chr.	Peak position (bp)	Block LD min	Block LD Max	QTL effect	LOD score	PEV (%)^b^	MAF^c^
HB	HB1.1	1, 4	Pv01	21,832,490	21,793,125	21,846,851	0.34	5.59	2.41	0.13
HB	HB1.2	1, 2, 3, 4, 5, 6	Pv01	44,984,153	44,808,951	45,071,760	−0.37	8.76	4.33	0.35
HB	HB3.1	2,3,5	Pv03	53,107,156	53,080,736	53,156,072	−0.34	6.29	3.43	0.38
HB	HB4.2	1,2,4,5,6	Pv04	541,783	528,814	544,841	0.80	8.75	3.30	0.03
HB	HB5.1	1,2,3,4,5	Pv05	39,065,428	38,872,419	39,106,281	−0.52	19.18	9.73	0.44
HB	HB8.1	1, 2	Pv08	62,009,281	61,984,614	62,015,035	0.30	6.89	2.99	0.25
HB	HB10.1	1, 6	Pv10	42,083,484	42,038,798	42,116,762	0.34	9.27	2.80	0.27

^a^mrMLM, FASTmrMLM, FASTmrEMMA, pLARmEB, pKWmEB, and EM-BLASSO were indicated by 1–6, respectively. ^b^PVE (%), percentage of phenotypic variation explained by each QTL. ^c^MAF, minor allele frequency determined from the peak significant SNP.

ADP121 (Kranskop HR-1) and the breeding line ADP723 (KAB06F2.8-69) developed by CIAT-Malawi, exhibited resistant HBB scores 1.7 and 2.7, respectively, and possessed the same haplotype with six QTL, HB1.1, HB3.1, HB4.2, HB5.1, HB8.1, and HB10.1. Moreover, ADP118 (Werna) and ADP716 (MW-1), a breeding line developed by CIAT-Malawi, with scores of 2.0 and 2.3, respectively, possessed the same haplotype with five QTL HB1.2, HB3.1/HB5.1, HB8.1, HB10.

### QTL intervals with resistance to multiple bacterial diseases

Five QTL intervals that were resistant to two or more bacterial pathogens were observed in Pv01 (BS1.1 and HB1.1), Pv01 (BS1.2, CB1.1, and HB1.2), Pv03 (BS3.1 and HB3.1), Pv05 (BS5.1 and HB5.1), and Pv08 (BS8.1 and HB8.1). The same peak SNP detected the QTL within the Pv01 (BS1.1 and HB1.1), Pv03, and Pv05 intervals, suggesting that a single gene likely contributes to resistance to both BBS and HBB bacterial pathogens.

The disease resistance conditioned by BS1.2, CB1.1, and HB1.2 within the second QTL interval on Pv01, was influenced by the *Fin* locus, located in the same region, which controls growth habit. For all three bacterial diseases, genotypes with indeterminate vine growth habits exhibited significantly (P <0.0001) higher resistance (lower susceptibility) than the genotypes with determinate bush growth habits (**Table 4**).

**Table 4:** ANOVA for quantitative BBS in Middelburg, CBB (Lusaka in 2014 and 2018), and HBB reaction (1 to 9 scale) in ADP accessions among bush, climber, and vine growth habits compared by Yuen’s trimmed means.

Trait	group1	n1	µ_trimmed_	group2	n2	µ_trimmed_	*P _Holm-adj._*	
**BS_Mburg**	Bush	202	4.74	Vine	120	2.96	0	****
**CBB_LskF_2014**	Bush	137	5.57	Vine	63	4.15	1.17E−06	****
**CBB_LskP_2014**	Bush	137	5.11	Vine	63	3.82	9.62E−09	****
**CBB_Lsk_2018**	Bush	176	6.43	Vine	77	4.52	1.33E−07	****
**HBB**	Bush	213	6.6	Vine	123	5.55	3.7E−07	****

****P <0.0001.

The peak SNPs for BS8.1 (S08_62072813) and HB8.1 (S08_62009281) were different, but they were in LD and were significantly correlated (D’ = 0.83; R^2^ = 0.58). Additionally, three QTL regions associated with CBB resistance were closely linked to QTL for resistance to BSS, HBB, or both diseases [BS5.1/HB5.1 (CB5.1), BS8.1/HB8.1 (SU91), and HB10.1 (SAP6)] (**Figure 3**; **Table 5**). The CB5.1 QTL, from 40,044,505 to 40,129,080 bp, was close to the above interval, but exhibited a low haplotype correlation (R^2^ = 0.036) with BS5.1 and HB5.1. The QTL overlapping the major SU91 QTL region from 62,534,585 to 62,555,715 bp exhibited low LD and correlation (D’ = 0.19; R2 = 0.015) with BS8.1 and HB8.1. The HB10.1 QTL, from 42,038,798 to 42,116,762 bp, was found to be moderately correlated (R^2^ = 0.46) with the SAP6 QTL, but their MAF values were quite different, 0.27 HB10.1 and 0.15 SAP6.

**Table 5:** Summary of candidate genes identified for five QTL intervals and three regions with linked QTL conditioning resistance to multiple bacterial pathogens BBS, CBB, and HBB.

QTL	Chromosome	Start (bp)	End (bp)	Candidate genes
**BS1.1/HB1.1**	Pv01	21,793,125	21,846,851	Phvul.001G103200, Phvul.001G103300, and Phvul.001G105101
**BS1.2/CB1.1/HB1.2**	Pv01	44,576,796	45,071,760	Phvul.001G188700 and Phvul.001G189200
**BS3.2/HB3.2**	Pv03	53,080,736	53,156,072	Phvul.003G294000 and Phvul.003G294200
**BS5.1/HB5.1**	Pv05	38,872,419	39,106,281	Cluster of 22 proteins with NB and LRR domains
**CB5.1**	Pv05	40,044,505	40,129,080	Phvul.005G175800
**BS8.1/H8.1**	Pv08	61,984,614	62,128,602	Cluster of five LRR proteins
**SU91**	Pv08	62,534,585	62,555,715	Niemann Pick transporter protein
**HB10.1**	Pv10	42,038,798	42,116,762	Cluster of 2 LRR proteins
**SAP6**	Pv10	40,991,447	41,152,975	Phvul.010G120401, Phvul.010G128900, Phvul.010G130500, Phvul.010130600, and Phvul.010G131400

## Discussion

The Andean diversity panel was examined using GWAS to identify regions conferring resistance to bacterial brown spot, common bacterial blight, and halo bacterial blight diseases, which can severely reduce yield and fitness in common bean. GWAS and disease evaluations represented a cooperative effort among common bean researchers across four countries: Puerto Rico, South Africa, the United States of America, and Zambia. Given that common beans represent a specialty crop with a relatively small research community, the increased scope and widened impact often result from collaborative research across continents on topics of mutual benefit.

We used mean BBS disease severity reactions for a subset of ADP evaluated by Salegua et al. (2020) under natural and artificial field inoculations in Middelburg and Potchefstroom, South Africa, respectively. Mean CBB disease reactions for subsets of ADP were obtained in this study under artificial screenhouse inoculations in Puerto Rico and Potchefstroom and natural field infections in Lusaka, Zambia, in 2014 and 2018. Mean HBB disease reactions for ADP were obtained from evaluations conducted under artificial field inoculation in Potchefstroom in a previous study (Tock et al., 2017). Although different subsets of ADP accessions were evaluated across these different trials, there were enough accessions (ranging from 99 to 323 accessions) to identify five QTL intervals with resistance to multiple bacterial pathogens and to observe significant correlations between disease reactions (1 to 9 severity scores) obtained for the three bacterial pathogens.

Overall, 24 QTL associated with resistance met our criteria for significance: i) significant in at least two of the six ML-GWAS methods conducted and ii) combined with significant Yuen’s tests. Zhang et al. (2019) suggested combining single-locus and/or multi-locus methods to enhance the detection power and robustness of GWAS. Roy et al. (2023) used multiple mapping methods and environments to increase confidence and reliability of identified QTL for use in marker-assisted selection.

Nine, eight, and seven QTL were associated with resistance to BBS, CBB, and HBB, respectively. Previous BBS QTL studies predated accurate physical maps such that the 21 QTL identified by Jung et al. (2003) across 10 of 11 linkage groups and the four QTL identified by Navarro et al. (2007) on chromosomes Pv01, Pv03, Pv06, and Pv11 could not be easily aligned with the nine QTL found herein. This study provides a starting point for physical mapping and naming of distinct BBS QTL.

There were sufficient differences in infection between the BBS trials, and only one QTL, BS5.1, was detected in both trials. Differences in climate, pathogen strains, mode of infection, and disease severity, among other factors, likely contributed to identification of distinct QTL under natural infection versus artificial field inoculation. Perhaps some of the BBS QTL detected by natural infection reduced the *Pss* population size on leaf surfaces, which lessened infection incidence and severity. Four BBS QTL overlapped with four HBB QTL, and one with both CBB and HBB QTL.

BS1.1 and HB1.1 were found linked to a cluster of three multi-antimicrobial extrusion (MATE) proteins, Phvul.001G103200, Phvul.001G103300, and Phvul.001G105101. In *Arabidopsis thaliana*, these proteins are highly expressed in the presence of the SAD2 gene, which encodes an importin β-like protein that plays a fundamental role in resistance against *P. syringae* pv. *tomato* (Li et al., 2023). Sun et al. (2011) identified and characterized an Activated Disease Susceptibility 1 (ADS1) gene, which encodes a putative MATE transport protein. They found that overexpression of ADS1 supported the increased growth of *P. syringae* pv. *phaseolicola*, which may control processes integral to the interaction between *Arabidopsis* and *P. syringae* species.

BS1.2, within the same QTL interval as CB1.1 and HB1.2, is co-located with the *Fin* locus that conditions growth habit. Accessions with determinate bush growth habits were clearly more susceptible to the three bacterial diseases than those with indeterminate vine growth habits, suggesting that *Fin* or a tightly linked gene has a pleiotropic effect on bacterial disease reactions. Early maturity and less plasticity for vegetative growth and the reproductive phase likely contribute to the increased susceptibility of accessions with determinate bush growth habits. Two gene models within the region, Phvul.001G188700 and Phvul.001G189200, affect agronomic traits including flowering time, vegetative growth, pod and seed size, and early seedling development (González et al., 2016; Moghaddam et al., 2016; Delgado-García et al., 2021). The Phvul.001G188700 gene model encodes a nucleoside hydrolase involved in mobilization of nutrients during plant development and response to plant–pathogen interactions, playing a role in manipulating plant metabolism to favor the pathogen (Li et al., 2013). Additionally, the Phvul.001G189200 gene, homologous to the *A. thaliana* TERMINAL FLOWER1 (TFL1) gene, has been suggested in previous studies as a candidate gene for the determinacy locus *fin* (Kwak et al., 2008; Repinski et al., 2012; Keller et al., 2022).

For the BS3.1 and HB3.1 QTL intervals, a significant SNP was found upstream of the gene model Phvul.003G294000, which encodes a peroxidase protein. Peroxidase FBP1 and RBOH oxidases play crucial roles in the defense mechanisms of *P. vulgaris* against *P. syringae* pv. *phaseolicola* (Galeou et al., 2023). Specifically, FBP1 has been implicated in rhythmic defense, whereas RBOH oxidases are associated with acute defense responses (Galeou et al., 2023). Another candidate gene, Phvul.003G294200, containing a Cyclophilin-type peptidyl-prolyl cis-trans isomerase domain, was associated with the plant defense response against *P. syringae* in *A. thaliana*, where knock-out mutations increased susceptibility to *P. syringae*, while overexpression altered the transcription of defense genes, enhancing resistance (Pogorelko et al., 2014).

BS5.1 and HB5.1 are associated with a cluster of 22 leucine-rich repeat (LRR) proteins. Oblessuc et al. (2022) observed that gene model Phvul.005G162600 (Pv05: 39.05 Mb), encoding a LRR/malectin protein, was significantly upregulated in HBB resistant and tolerant genotypes, suggesting a potential association with general plant responses to *Psph*. LRR/malectin are plasma membrane receptors frequently found in legume and non-legume species (Restrepo-Montoya et al., 2020), and are involved in pathogen resistance and antimicrobial responses (Wang et al., 2008; Chakraborty et al., 2019).

For the BS8.1 and HB8.1 interval, Oblessuc et al. (2022) identified two candidate genes, Phvul.008G277310 and Phvul.008G277352 for resistance to *Psph*, at 61.9 Mb, which encode Ras suppressor proteins containing leucine-rich repeats. Overall, the candidate gene models found within the intervals with overlapping QTL suggested genes that could confer resistance to both BBS and HBB.

For CBB, two well-known major effect QTL, SAP6 and SU91, were detected. Both SAP6 and SU91 are easily assayed with existing markers in an Andean genetic background based on their origin from the Middle American gene pool and Tepary bean, respectively. Normally, SU91 is detectable under controlled screening conditions, but few accessions with this QTL were included in screenhouse trials. Therefore, SU91 was only detected in the field and exhibited a low MAF (0.03) because few accessions possessed the QTL (RH No.21, OPS-RS4, Werna, USDK-CBB-15, and USWK-CBB-17). Simons et al. (2021) identified SU91 within the 62.39–62.40 Mb interval in a Middle American breeding population. Viteri et al. (2014) mapped the peak for SU91 QTL at 62.96 bp (updated to G19833v2.1) in the Othello/VAX 3 RIL population. Lobaton et al. (2018) also detected an introgression block from *P. acutifolious* from 62,536,519 to 63,032,528 bp in the interspecific lines VAX 3, VAX 4, and VAX 6. Perry et al. (2013) identified a candidate gene at 62.9 Mb linked to the SU91 QTL, which encodes a Niemann–Pick transporter protein.

Across the different CBB trials, the major QTL SAP6 was identified within an interval of 40,991,447–41,152,975 bp. GWAS in a panel of 852 dry bean inoculated with *Xap* f91-5 strain located SAP6 QTL to 41.66–41.84 Mb in a Middle-American breeding population and to 41.11–42.22 Mb in an Andean breeding population (Simons et al., 2021). Zhu et al. (2016) described 25 gene models for the SAP6 region spanning 40.83–41.11 Mb (updated to G19833 v2.1). Among these gene models, 10 have functional annotations associated with plant–pathogen interactions, including receptor-like kinases, lipoxygenase, cytochrome P450 superfamily members, and plant glycoproteins from the cupin superfamily.

The specific candidate genes for SAP6 included four encoding MYB transcription factors (Pv010G131400, Pv010G120401, Pv010G130500, and Pv010130600). In Arabidopsis, AtMYB30, a MYB TF, has been implicated as an activator of HR-related cell death and resistance against the bacterial pathogen *X. axonopodis* (Celenza et al., 2005). Additionally, another candidate gene, Phvul.010G128900, encodes a Cytochrome P450 superfamily protein, similar to the pepper Cytochrome P450 gene CaCYP450A, which is differentially induced during *Xanthomonas campestris* pv. *vesicatoria* infection, and plays a crucial role in plant defense (Hwang and Hwang, 2010).

Except for SAP6, different QTL conditioning resistance to CBB were identified in the field (three QTL) and screenhouse (four QTL) screening environments. The low MAF (0.01 to 0.04) for the CB5.1, CB6.1, CB10.2, and CB11.4 QTL detected in the screenhouse suggest a potential origin from somewhere other than the Andean gene pool, and these rare alleles require further validation. The CB5.1 QTL interval is associated with the candidate gene Phvul.005G175800, a pectin lyase-like superfamily protein. In a previous study, a QTL was found linked to the Bng162 marker at 40.45 Mb (updated to G19833v2.1) in a F_2:4_ population (Seaforth/OAC95) evaluated by artificial CBB inoculation under field conditions (Yu et al., 2000; Tar’an et al., 2001). Kraiselburd et al. (2013) observed differential expression of genes encoding cell wall modification-related proteins in *Citrus sinensis* inoculated with *Xanthomonas citri* subsp. *citri*. CB6.1, with a large effect (1.74, 2.09) and high LOD (35.5, 16.6) for resistance to Xa3353 and Xa484A strains, respectively, was linked (0.16 Mb) downstream of the desirable *bc-3* recessive gene, which is resistant to all BCMV and BCMNV strains. The gene model, Phvul.010G022400, linked to QTL CB10.2, was found through a transcriptomic analysis conducted on HR45 (Yang et al., 2022), a genotype known for its high resistance to *Xap*. This gene encodes a cytochrome P450 enzyme associated with oxidation–reduction processes.

There have been concerted traditional breeding efforts in the past to move the resistance conditioned by SAP6 QTL, which is supported by its higher MAF (0.15–0.18). This is compared to the recent movement of SU91 into Andean beans, as evidenced by the lower MAF of 0.03 in one trial and the complete lack of detection in the other trials. The use of different strains in the screenhouse trials may have contributed to the detection of five QTL in Puerto Rico compared to SAP6 alone in South Africa. Although the Xa3353 strain is more aggressive than the Xa484A strain, they both detected the same five QTL in the Puerto Rico trials, which suggests that using only one of these strains may be sufficient for screening the germplasm for CBB resistance. Xa484A was originally used in the discovery of SAP6 QTL (Miklas et al., 1996).

In addition to SAP6 and SU91, CB1.1 and CB7.1 were detected in field trials. CB1.1 is associated with growth habits. CB7.1, which exhibited a large MAF (0.32–0.48) and was detected in only one trial (Lusaka, 2014), which suggests that it may be influenced by a common background gene that is environmentally sensitive. Two receptor-like kinase (RLK) genes (Phvul.007G051300 and Phvul.007G030300) found in the CB7.1 region in a transcriptomic study were downregulated in JaloEEP558, a genotype susceptible to *Xap*, in contrast to BAT93, which exhibited minimal symptoms (Foucher et al., 2020).

Seven QTL identified as conditioning HBB resistance to *Psph* Race 6 in this study compared to only HB5.1 detected by Tock et al. (2017). HB4.2 and HB5.1 were also detected in RIL populations by Tock et al. (2017). Oblessuc et al. (2022) analyzed two candidate genes in proximity to HB4.2, Phvul.004G008740 and Phvul.004G015800, both with LRR domains, which were significantly upregulated in genotypes tolerant to the *Psph* strain but downregulated in resistant genotypes. Besides HB1.1 and HB4.2, the large MAF (0.25 to 0.44) for HB1.2, HB3.1, HB5.1, HB8.1, and HB10.1 suggests they represent background genes that influence HBB disease severity. HB10.1 is near the *Pse-4* gene located in an interval from 40.69 – 41.08 Mb (updated to G19833v2.1), which conditions resistance to Race 5 (Miklas et al., 2014). Two candidate genes, Phvul.010G136700 and Phvul.010G136800, were identified for HB10.1, which encode LRR proteins. Five HBB QTL overlapped with QTL conditioning resistance to BBS, as described above.

In summary, multiple regions associated with quantitative resistance to *Pseudomonas* and *Xanthomonas* were identified in Andean beans. Four QTL contributing to resistance to both BBS and HBB were found in Pv01, Pv03, Pv05, and Pv08. Growth habits likely contribute to the QTL interval on Pv01, which influences field reactions to all three bacterial diseases evaluated in this study. BS5.1, detected under both natural infection and artificial field inoculation, and SAP6, identified in all CBB trials, represented QTL with stable and broad effects. QTL HB5.1, CB7.1, and CB10.2 were further validated by their overlap with candidate genes reported in previous transcriptomic studies in common bean. Some QTL with large effects BS1.1 (0.6), CB6.1 (1.74 and 2.09), and CB11.4 (1.10, 1.68) represent potential targets to combine with major QTL HB5.1, SAP6, SU91, HB4.2, and HB5.1, to increase resistance to BBS, CBB, and HBB.

## Data Availability

The authors acknowledge that the data presented in this study must be deposited and made publicly available in an acceptable repository, prior to publication. Frontiers cannot accept a article that does not adhere to our open data policies.
